# pCO2 values in asphyxiated infants under therapeutic hypothermia after tailored respiratory management: a retrospective cohort study

**DOI:** 10.3389/fped.2023.1293526

**Published:** 2024-01-23

**Authors:** Francesca Serrao, Eloisa Tiberi, Tommaso Verdolotti, Domenico Marco Maurizio Romeo, Mirta Corsello, Elisa Pede, Francesco Cota, Simonetta Costa, Francesca Gallini, Cesare Colosimo, Eugenio Maria Mercuri, Giovanni Vento

**Affiliations:** ^1^ Division of Neonatology, Department of Women, Children and Public Health Sciences, University Hospital Fondazione Policlinico Universitario Agostino Gemelli IRCCS, Rome, Italy; ^2^ Radiology and Neuroradiology Department, Fondazione Policlinico Universitario Agostino Gemelli IRCCS, Catholic University, Rome, Italy; ^3^ Pediatric Neurology Unit, Fondazione Policlinico Universitario Agostino Gemelli IRCCS, Catholic University, Rome, Italy

**Keywords:** perinatal asphyxia, hypoxic ischemic encephalopathy, therapeutic hypothermia, mechanical ventilation, hypocapnia

## Abstract

**Background:**

Hypoxic-ischemic encephalopathy (HIE) represents one of the major causes of neonatal death and long-term neurological disability. Both hypoxic-ischemic insults and therapeutic hypothermia (TH) can affect respiratory function. Currently, there is no evidence regarding optimal respiratory management in these infants.

**Methods:**

This is a retrospective cohort study examining newborns with HIE treated with TH between January 2015 and September 2020. The study population was divided into two groups based on different respiratory assistance during TH: spontaneous breathing (Group A) or mechanical ventilation (Group B). The primary outcome of the study was the mean pCO_2_ ± SD evaluation during TH in ventilated and non-ventilated asphyxiated infants. The secondary outcome was the correlation between ventilation strategy and short-term neurologic outcome according to Rutherford et al.'s MRI scoring system.

**Results:**

A total of 126 newborns were enrolled, 75 in Group A and 51 in Group B. Respiratory management was individualized, and volume guarantee (VG) ventilation was the first choice for ventilated infants. Group B infants showed more severe conditions at birth. During TH, ventilated infants showed optimal mean pCO_2_ comparable with those breathing spontaneously (40.6 mmHg vs. 42.3 mmHg, respectively, *p* 0.091), with no significant difference in pCO2 standard deviation between (7.7 mmHg vs. 8.1 mmHg, respectively, *p* 0.522). Mean pH, pH standard deviation, mean pO_2_, pO_2_ standard deviation, and mean respiratory rate also did not differ between groups. MRI patterns of brain injury predictive of abnormal neurodevelopmental outcomes were similar in both groups. Logistic regression analysis demonstrated that only umbilical cord arterial blood pH-affected MRI lesions were associated with poor neurodevelopmental outcomes (OR 1.505; CI 95% 1.069–2.117).

**Conclusions:**

Infants cooled after HIE should receive individualized respiratory management, not necessarily involving intubation. In those infants requiring mechanical ventilation, a volume-targeted strategy appeared to be effective in maintaining stable blood gas levels. Short-term neurological outcomes appeared comparable in ventilated and non-ventilated infants.

## Introduction

Birth asphyxia is a major cause of neonatal death and long-term neurological sequelae worldwide. It is characterized by impaired blood gas exchange, which, if persistent, leads to progressive hypoxemia and hypercapnia with significant metabolic acidosis (1). The severity of the subsequent multi-organ involvement is the result of a complex balance between the degree, duration, and type of asphyxia and the quality of the cardiovascular compensatory response (2).

Hypoxic-ischemic encephalopathy (HIE) is the expression of brain damage resulting from asphyxia and accounts for 23% of all neonatal deaths worldwide (3).

Therapeutic hypothermia (TH) has been shown to be effective in improving the outcome of these neonates by reducing the risk of death and disability (4). Mechanical ventilation is often necessary as a supportive therapy for severely asphyxiated infants (5) who may have various causes and degrees of respiratory failure: meconium aspiration syndrome, pneumonia, pulmonary hemorrhage, surfactant deficiency, air leaks, pulmonary hypertension, lack of spontaneous respiratory activity due to severe neurological impairment, or seizures (6). Both asphyxia and TH itself can impair pulmonary function and gas exchange. Understanding how hypothermia affects respiratory function and lung mechanics is particularly important in order to apply “neuroprotective” ventilation strategies to avoid secondary brain damage associated with hyperventilation and cerebral vasoconstriction (7).

Infants with HIE, especially those undergoing TH, have a decreased basal metabolic rate, decreased brain energy utilization, and carbon dioxide production, and tend to hyperventilate to compensate for metabolic acidosis; therefore, hypocapnia could be considered a common phenomenon in these infants. A link between hypocapnia and brain damage has already been demonstrated in animal models, in preterm infants, and in infants with HIE. Patients with a lower minimum pCO2 and a higher cumulative pCO2 <35 mmHg showed a worse neurological outcome at 18 months of age; moreover, low pCO2 values were inversely and independently associated with an unfavorable outcome (9–11). Intubated and ventilated asphyxiated neonates appear to have more severe hypocapnia and a higher incidence of brain damage (12).

In this population, it is more important to avoid both hypo- and hypercarbia: the former may cause cerebral vasoconstriction, leading to decreased cerebral blood flow with a higher risk of ischemic events and seizures; the latter has the opposite effect, increasing cerebral blood flow and thus exacerbating reperfusion injury (13).

The primary outcome of this study was the mean ± SD pCO2 assessment during TH in ventilated and non-ventilated asphyxiated infants.

The secondary outcome was the correlation between the ventilation strategy used during TH and short-term neurological outcomes.

## Methods

This retrospective cohort study examined newborns with HIE and treated with TH between January 2015 and September 2020 at the Neonatal Intensive Care Unit (NICU) of the Fondazione Policlinico Universitario A. Gemelli IRCCS, Rome, Italy. TH was initiated within 6 h of life in infants with gestational age ≥35 weeks and birth weight ≥1,800 g if the following two criteria were met: (1) intrapartum hypoxia with at least one of the following: (A) Apgar score ≤5 at 10 min; OR (B) persistent need for resuscitation at 10 min; OR (C) blood gas acidosis characterized by either pH ≤7.0 or negative base excess ≥12 mmol/L in the first hour of life; (2) moderate to severe encephalopathy evaluated between 30 and 60 min after birth (14). Neurological involvement was confirmed by an amplitude-integrated electroencephalogram (aEEG) defined as moderately abnormal with an upper margin >10 µvolts and lower margin <5 µvolts or severely abnormal with an upper margin <10 µvolts and lower margin <5 µvolts or burst suppression.

Patients were excluded if they received a diagnosis of congenital malformation or encephalopathy of another cause. Outborn neonates began passive cooling at the transferring center, avoiding heating and aiming for a body temperature of 35°C. The target rectal temperature was 33.5°C (± 0.5°C), obtained with the CritiCool ® system and maintained by servo-controlled temperature monitoring and regulation; TH was prolonged for 72 h, followed by slow rewarming with gradual increases of no more than 0.5°C per hour, until normothermia was reached.

Daily clinical care for the enrolled patients was provided by the attending physician in accordance with usual practice. Pregnancy pathologies were defined as metabolic disease (diabetes or hepatosis), infectious disease (chorioamnionitis, rupture of membranes >18 h, fever, vaginal, or rectal colonization by group B beta-hemolytic streptococcus), and cardiovascular disease (gestational hypertension or pre-eclampsia).

Perinatal asphyxia sentinel events such as fetal bradycardia, pathologic cardiotocography, stained amniotic fluid, placental abruption, cord prolapse, reduced fetal movements, and shoulder dystocia were also collected. During hypothermia, patients received routine clinical care, including monitoring of vital signs and surveillance for organ dysfunction. An umbilical venous catheter was positioned; peripheral venous lines and umbilical arterial catheters were placed when needed.

Respiratory management was individualized according to the patient's clinical status and possible overlapping pulmonary diseases. Tailored ventilation allowed for the treatment of respiratory insufficiency and the prevention of hyperoxia and hypocapnia. Intubation and ventilation were initiated in the delivery room in the most severe cases or in the NICU on the basis of excessive or absent respiratory effort (Silverman score >6 or absent/irregular spontaneous breathing), a fraction of inspired oxygen (FiO_2_) ≥35% during non-invasive ventilation, hypercapnia >60 mmHg for longer than 30 min, recurrent apneic crisis not resolved with non-invasive ventilation, or progressive respiratory acidosis. On the other hand, hypocapnia was not considered a criterion for the initiation of mechanical ventilation.

Synchronized Intermittent Mandatory Ventilation with volume guarantee (SIMV + VG) was the first choice, while High-Frequency Oscillatory Ventilation with volume guarantee (HFOV + VG) was chosen in the case of air leak syndrome or meconium aspiration syndrome with pulmonary hypertension.

The goals of respiratory management were pH >7.25, pCO_2_ 40–55 mmHg, pO_2_ 50–70 mmHg, and SpO_2_ 92%–95%. In order to maintain these parameters, the initial ventilatory settings were as follows: tidal volume (VT) 4 ml/kg, positive end-expiratory pressure (PEEP) 4 cmH_2_O, inspiratory time (Ti) 0.4 s, respiratory rate (RR) 40 breaths/minute in SIMV-VG modality; VT_hf_ 2 ml/kg, mean airway pressure (MAP) 10 cmH_2_O, frequency 7–10 Hz, inspiration to expiration ratio of 1:1 in HFOV-VG modality. Nasal Continuous Positive Airway Pressure (nCPAP) and nasal Intermittent Positive Pressure Ventilation (nIPPV) were used as non-invasive ventilation modalities as the primary mode or post-extubation. Some patients required only oxygen enrichment. The decision to initiate Inhaled Nitric Oxide (iNO) therapy was based on an echocardiogram and/or oxygenation index suggestive of pulmonary hypertension. Surfactant administration was reserved for newborns with a diagnosis of respiratory distress syndrome, meconium aspiration syndrome, pulmonary hemorrhage or pneumonia, and FiO_2_ ≥0.35.

All newborns received personalized parenteral nutrition, starting with an overall liquid supply of 50–55 ml/kg and considering the possible presence of dyselectrolytemia, acute kidney injury, and the syndrome of inappropriate antidiuretic hormone secretion (SIADH). Seizures were treated with phenobarbital as a first-line drug, if not effective with midazolam. Broad-spectrum antibiotic prophylaxis was used routinely, with modification or initiation of targeted therapy in cases of known maternal or neonatal culture positivity. Pharmacological cardiovascular support was modulated based on functional echocardiographic assessments performed within the first 24 h of life.

Pain assessment and management included: serial evaluation using the NPASS score; minimization of the number of painful stimuli; sedation with opioids administered continuously through a dedicated line at the minimum necessary dose (starting with 0.075 µg/kg/min of remifentanil); and additional bolus (e.g., fentanyl, 2 µg/kg). Early enteral feeding by nasogastric tube for trophic purposes was given to all infants in the absence of abdominal pathology or inotropic support.

The severity of neonatal encephalopathy was assessed by the modified Sarnat score^14^ within 1 hour of birth, either at the transferring center or on admission. Neurological function was monitored by continuous aEEG within the first 6 h of age and throughout the entire 72-h TH period; after rewarming, an electroencephalogram was performed and reviewed by an experienced pediatric neurologist; and magnetic resonance imaging (MRI) was performed between days 7 and 10 after birth. Neuroimaging results were evaluated according to Rutherford et al. (15, 16). Moderate or severe lesions in the basal ganglia and thalamus, an abnormal posterior limb of the internal capsule, or severe white matter lesions were deemed to be predictive of an abnormal neurodevelopmental outcome (17).

The study protocol was approved by the Ethics Committee of the Fondazione Policlinico Universitario A. Gemelli IRCCS, Rome, Italy, with the approval number 12692/22 ID 4867.

## Data collection

Ventilator data were collected from ventilation sheets, where doctors in our unit usually report vital signs and respiratory and ventilation parameters in newborns receiving respiratory support. Information was collected on FiO_2_, VT per weight, RR, and total minute ventilation, including spontaneous and mechanical breaths. Capillary blood samples for gas analysis were obtained after adequate heel warming at least every 12 h from birth through the duration of TH. Results were temperature-corrected to 37°C using a blood gas analyzer. Hypocapnia was defined as pCO2 less than 35 mmHg (6). For each patient, we calculated the mean ± SD of six values of all parameters of interest obtained every 12 h during TH from blood gas analysis and respiratory sheets.

## Statistical analyses and sample size

Results are shown as mean ± standard deviation for continuous variables and as absolute numbers and percentages for dichotomous variables. Statistical analyses were performed using the Wilcoxon rank-sum test (Mann–Whitney U Test), Student's *t*-test, Saphiro–Francia *W*-test, and Fisher's exact test. A *p*-value of <0.05 was considered statistically significant.

The sample size calculation was based on our observational data showing that the mean ± standard deviation (SD) pCO_2_ in non-ventilated asphyxiated infants undergoing TH was 40 ± 6 mmHg.The sample size calculation indicated that at least 23 patients in each group were needed to detect a 5 mmHg difference in ventilated asphyxiated infants with 80% power and a 0.05 alpha error. Analyses were performed using STATA/IC15.

## Results

During the study period, 126 newborns met the inclusion criteria; the population was divided into two groups based on different respiratory assistance during TH: spontaneous breathing (Group A) or mechanical ventilation (Group B). The general characteristics of the two study groups are shown in Table 1.

**Table 1:** General characteristics of the two study groups.

	Spontaneous breathing group	Mechanical ventilation	*p*-value
	*N* = 75	*N* = 51	
Gestational age (weeks)	39.4 ± 1.3	39.0 ± 1.8	0.17
Birth weight (g)	3,318 ± 474	3,303 ± 667	0.87
Male patients, *n* (%)	54 (72)	29 (57)	0.088
Cesarean section, *n* (%)	22 (29)	20 (39)	**0**.**001**
Outborn, *n* (%)	57 (76)	31 (61)	0.078
Small for gestational age, *n* (%)	10 (13)	6 (12)	1.00
Large for gestational age, *n* (%)	9 (12%)	6 (12)	1.00
1′ APGAR	4.3 ± 2.0	2.9 ± 2.3	**0**.**0002**
5′ APGAR	6.6 ± 1.8	4.8 ± 2.2	**0**.**0000**
10′ APGAR	7.3 ± 1.8	5.6 ± 2.2	**0**.**0000**
Arterial umbilical cord blood Ph	7.05 ± 0.15	7.00 ± 0.19	**0**.**0095**
Arterial umbilical cord base deficit (mmol/L)	−16.5 ± 4.9	−16.8 ± 5.7	0.839
Maternal metabolic disease, *n* (%)	16 (21)	10 (20)	1.000
Maternal infectious disease, *n* (%)	5 (7)	15 (29)	**0**.**001**
Maternal cardiovascular disease, *n* (%)	6 (8)	2 (4)	0.472
Perinatal asphyxia sentinel events, *n* (%)	19 (25)	28 (55)	**0**.**001**
Mild HIE, *n* (%)	4 (5)	1 (2)	**0**.**002**
Moderate HIE, *n* (%)	66 (88)	36 (71)	
Severe HIE, *n* (%)	4 (5)	14 (27)	
Moderately abnormal aEEG, *n* (%)	53 (71)	27 (53)	0.071
Severely abnormal aEEG, *n* (%)	12 (16)	15 (29)	
Burst Suppression aEEG, *n* (%)	2 (3)	5 (10)	

Values are expressed as the mean ± SD, or number (%). *P*-values <0.05 were considered to be statistically significant. HIE, Hypoxic-ischemic encephalopathy; aEEG, amplitude-integrated electroencephalogram.

Newborns in Group B had a more severe overall condition at birth than those in Group A, with significantly lower Apgar scores at 1, 5, and 10 min, lower umbilical blood cord pH, and a higher percentage of severe encephalopathy. In the pregnancy history, the prevalence of maternal metabolic and cardiovascular diseases was similar in both groups, whereas Group B had more maternal infectious diseases (29% vs. 7%, *p *= 0.001) and acute perinatal sentinel events (55% vs. 25%, *p* = 0.001).

Neurological examination between 30 and 60 min after birth showed severe HIE more frequently in group B (*p* = 0.002).

On the other hand, no statistically significant differences were identified in the aEEG pattern (*p* = 0.071).

Table 2 shows the prevalence of major pulmonary pathologies and respiratory characteristics of the patients.

**Table 2:** Pulmonary pathologies and respiratory characteristics of two study groups during TH.

	Spontaneous breathing	Mechanical ventilation	*p*-value
	*N* = 75	*N* = 51	
RDS requiring surfactant therapy	2 (2.7%)	21 (41.2%)	**0**.**000**
Pneumonia	1 (1.3%)	8 (15.7%)	**0**.**003**
Pulmonary hypertension	0 (0%)	10 (19.6%)	**0**.**000**
Meconium aspiration syndrome	0 (0%)	6 (11.7%)	0.023
Pneumothorax	0 (0%)	4 (7.8%)	**0**.**025**
Days of mechanical ventilation	0 ± 0	5.3 ± 11.7	**0**.**0001**
Days of non-invasive ventilation	9 (12.05)	16 (31.4)	0.11
Days of oxygen therapy	14 (18.7%)	36 (70.6%)	**0**.**000**
Ph	7.31 ± 0.42	7.33 ± 0.04	0.639
Ph SD	0.10 ± 0.45	0.05 ± 0.02	0.451
pCO2	40.6 ± 6.0	42.3 ± 5.1	0.091
pCO_2_ SD	7.7 ± 3.7	8.1 ± 3.9	0.522
pCO_2_ < 35 mmHg	13 (17.3)	5 (9.8)	0.304
pO_2_	58.9 ± 14.4	63.7 ± 20.7	0.135
pO_2_ SD	20.9 ± 13.1	20.7 ± 11.9	0.944
Base deficit	−2.2 ± 2.5	−3.1 ± 3.8	**0**.**0462**
Base deficit SD	3.4 ± 1.8	3.2 ± 1.9	0.460
Mean respiratory rate	48.8 ± 10.9	45 ± 12.6	0.087
Respiratory rate SD	10.3 ± 8.8	10.4 ± 7.1	0.915

Values are expressed as the mean ± SD, or number and %. *P*-values <0.05 were considered to be statistically significant. RDS, respiratory distress syndrome.

Respiratory distress syndrome requiring surfactant administration was diagnosed in 21 (41.2%) newborns in Group B and in 2 (2.7%) patients in Group A (*p* = 0.000). The IN-SUR-E technique for surfactant administration was used in spontaneously breathing infants. Pneumonia occurred in 8 (15.7%) newborns in Group B and in one patient (1.3%) in Group A (*p*-value = 0.003).

Pulmonary hypertension, pneumothorax, and meconium aspiration syndrome occurred only in newborns in Group B (*p* = 0.000, *p* = 0.025 and *p* = 0.023, respectively).

The mean duration of ventilation in group B patients was 5.3 ± 11.7 days.

**Figure 1:**
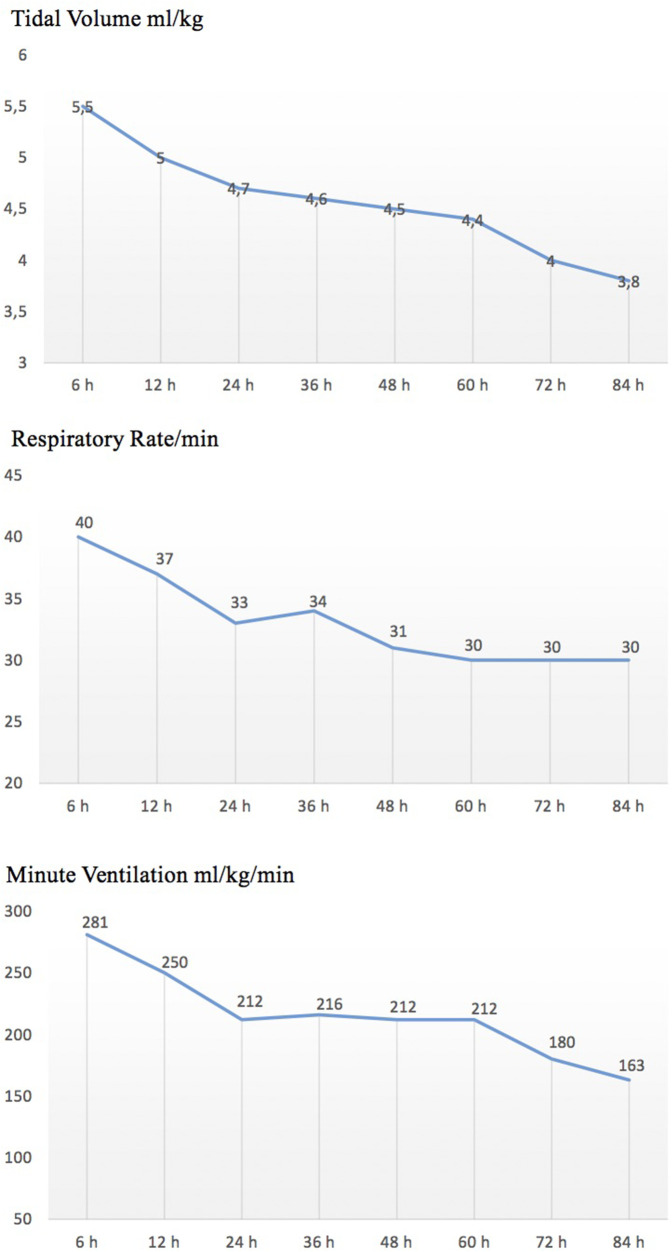
Trend of ventilator settings in patients undergoing SIMV-VG during therapeutic hypothermia.

SIMV + VG ventilation was used in 45 (88%) newborns, while HFOV + VG ventilation was used in 6 (12%) newborns. Data on VT, minute ventilation, and ventilator-set RR in patients undergoing SIMV + VG during 72 h of TH are shown in Figure 1.

All 6 newborns ventilated in the HFOV + VG modality were affected by meconium aspiration syndrome, complicated by pulmonary hypertension.

Infants in Group B had a longer duration of oxygen therapy.

**Figure 2:**
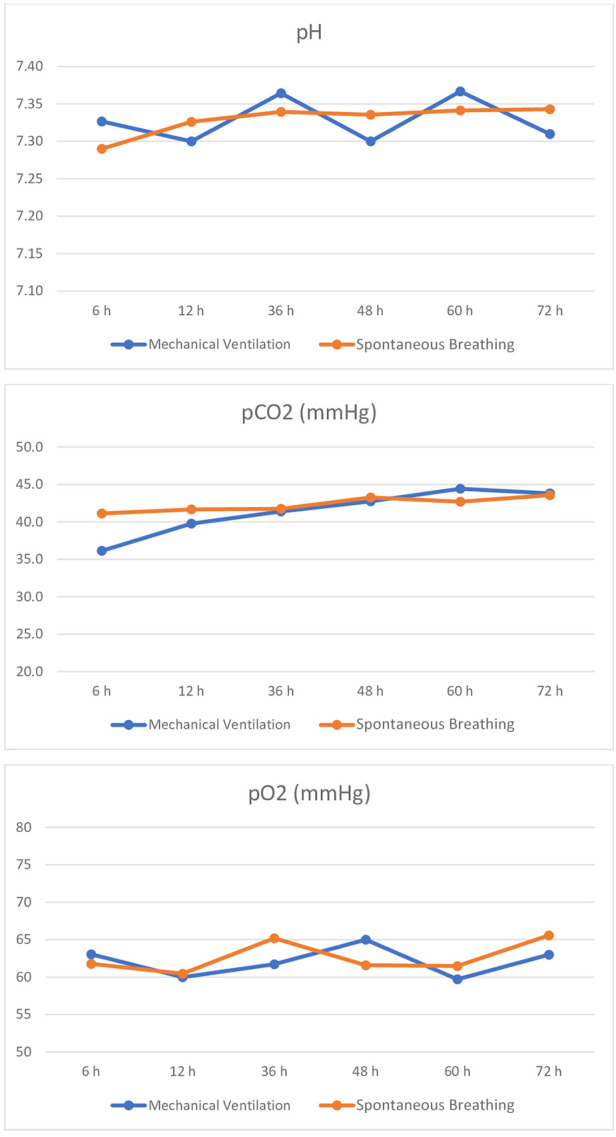
Trend of pH, pCO2, and pO2 during TH in spontaneously breathing and mechanically ventilated infants.

During TH, mean pH, pH standard deviation, mean pCO_2_, pCO_2_ standard deviation, mean pO_2_, pO_2_ standard deviation, mean RR, and RR standard deviation did not differ between the two groups. The trend of pH, pCo2, and pO2 values during TH is shown in Figure 2.

The mean value of base excess was more negative in newborns in Group B (*p* = 0.04).

Infants with mean pCO_2_ values below 35 mmHg were similar in the two groups (*p* 0.304).

The two groups had a significant difference in the incidence of hypotension (Group A 5.3%, Group B 29.1%, *p* = 0.000), anemia (Group A 8.0%, Group B 29.1%, *p* 0.003), thrombocytopenia (Group A 2.7%, Group B 19.6%, *p* 0.003), bleeding disorders (Group A 6.7%, Group B 37.3%, *p* 0.000), and late-onset sepsis (Group A 2.7%, Group B 13.7%, *p* = 0.030).

The two groups did not differ in glycemic disorders, renal failure, adiponecrosis, or urinary tract infections.

We observed a longer need for parenteral nutrition in Group B (9.2 vs. 4.8 days, *p* 0.000) and consequently later full enteral feeding (12 vs. 7 days, *p* 0.000). Group B showed a prolonged hospital stay (24.5 vs. 14.3 days, *p* 0.038).

Table 3 shows the short-term neurological outcome data; Group B has a higher incidence of seizures (*p* 0.001).

**Table 3:** Short-term neurological outcomes.

	Spontaneously breathing	Mechanical ventilation	*p*
	*N* = 75	*N* = 51	
Seizures	11 (14.7%)	21 (41.2%)	**0**.**001**
Magnetic resonance (days)	9.1 ± 2.8	12.4 ± 4.8	**0**.**000**
BGT normal	63 (84%)	33 (64.7%)	0.061
BGT mild	6 (8%)	7 (13.73%)	
BGT moderate	5 (6.7%)	7 (13.7)	
BGT severe	1 (1.3%)	4 (7.84%)	
PLIC normal	65 (86.7%)	35 (68.6%)	**0**.**040**
PLIC equivocal	8 (10.7)	11 (21.6%)	
PLIC abnormal	2 (2.7%)	5 (9.8%)	
WM normal	51 (68.0%)	37 (72.5%)	0.635
WM mild	3 (4.0%)	1 (1.9%)	
WM moderate	19 (25.3%)	10 (19.6)	
WM severe	2 (2.7%)	3 (5.8)	
Cortex normal	64 (85.3%)	44 (86.2%)	**0**.**019**
Cortex mild	9 (12.0%)	1 (1.9%)	
Cortex moderate	0 (0%)	0 (0%)	
Cortex severe	2 (2.6%)	6 (11.8%)	
Hemorrhage	49 (65.3%)	29 (56.9)	0.356
Lesions predictive of abnormal outcome	6 (8.0%)	10 (19.61)	0.06

Values are expressed as the mean ± SD, or number and %. *P*-values <0.05 were considered to be statistically significant. BGT, basal ganglia and thalami; PLIC, posterior limb of the internal capsule; WM, white matter.

The MRI pattern of brain injury predictive of abnormal neurodevelopmental outcome, according to Rutherford et al.'s (15, 16) scoring system, was similar in both groups, but lesions in the posterior limb of the internal capsule and the cortex only were significantly more represented in Group B (*p* = 0.040 and 0.019, respectively).

Logistic regression analysis (Table 4) demonstrated that umbilical cord arterial blood pH only affected the odds of MRI lesions related to poor neurodevelopmental outcome (OR 1.505; CI 95% 1.069–2.117). Instead, mechanical ventilation and the mean pCO_2_ blood gas level during TH did not affect the MRI pattern.

**Table 4:** Logistic regression analysis of risk factors for MRI lesions related to poor neurodevelopmental outcome.

Variable	Odds ratio (95% CI)	*p*
Arterial umbilical cord blood Ph	1.51 (1.07–2.12)	**0**.**019**
Mechanical ventilation	2.50 (0.74–8.50)	0.142
pCO2	0.94 (0.83–1.05)	0.296
pCO2 SD	0.985 (0.84–1.16)	0.860
pO2	0.99 (0.95–1.03)	0.582
pO2 SD	1.00 (0.94–1.05)	0.868

*P*-values <0.05 were considered to be statistically significant.

In the multivariate logistic regression analysis, lower 5-min Apgar score (OR 0.63 95% 0.486–0.813; *p* 0.000), intrapartum sentinel events (OR 3.805; 95% CI 1.03–10.316; *p* = 0.010), maternal infectious disease (OR 6.977; 95% CI 1.941–25.073; *p* = 0.003), cesarean section (OR 4.311; 95% CI 1.564–11.881; *p* = 0.005), and hypotension (OR 5.054; 95% CI 1.263−27.123; *p* = 0.024) were associated with the need for mechanical ventilation.

## Discussion

The use of TH is an effective and safe treatment for HIE neonates and has led to a reduction in mortality and severe neurological disability over time. Optimization of neonatal intensive care during TH may prevent the progression of injury, although there is no evidence regarding optimal ventilatory management in the literature. Previous studies have shown an association between hypocapnia and poor neurological outcomes (9–11). Adverse outcomes appear to be related not only to hypocapnia itself but also to cumulative exposure to hypo/hypercarbia and to pCO2 variability (18). In addition, lower carbon dioxide levels have been found in ventilated patients (12).

The present study describes a population of 126 neonates treated with TH and compares the clinical and laboratory characteristics and short-term neurological outcomes of mechanically ventilated and spontaneously breathing neonates. Ventilation management was optimized according to clinical conditions. Infants who required mechanical ventilation had worse clinical birth conditions compared to spontaneously breathing infants in terms of umbilical arterial pH, Apgar score, intrapartum events, and neurological examination in the first hour. They were also born to mothers with a significantly higher incidence of infectious diseases, which could explain their significantly higher incidence of congenital pneumonia. This could indicate an earlier onset of the hypoxic event, as previously hypothesized (19).

Patients ventilated during TH differed from spontaneously breathing infants not only by the occurrence of respiratory failure, but also by various comorbidities, such as hypotension requiring medical treatment, anemia, coagulopathy, thrombocytopenia, seizures, and pneumonia. This is associated with a predictable delay in achieving full enteral nutrition and prolonged hospitalization.

These observations are consistent with other studies in which the need for ventilation was associated with a worse overall clinical status.

Hypocapnia has been shown to be an independent variable that increases the risk of death and unfavorable neurodevelopmental outcomes. Hypocapnia may exacerbate brain injury by impairing cerebral blood flow, cellular energy metabolism, oxygen transport and oxygen extraction, and clearance of neurotoxic metabolites (8). Acute changes in pCO2 could be particularly detrimental to a brain with impaired cerebral blood flow regulation, as would be expected in HIE.

Thus, if we consider hypocapnia not only as an effect of severe birth asphyxia but also as a modifiable risk factor for secondary brain damage, we should strive to avoid both extreme carbon dioxide levels and their fluctuations in sicker patients altogether.

Several studies have reported that the management of ventilation during TH is very difficult and requires many complex adjustments and monitoring to avoid hyperventilation (20).

The lack of guidelines for ventilation strategies during TH leads to great variability in management between centers (21). This may explain why, in many centers, all infants are intubated and sedated during TH.

The ventilated infants in our study had optimal mean pCO2 values that were comparable to those of spontaneously breathing infants. These results could be due to the volume-controlled ventilation used on patients. However, this observation should be taken with caution, as different modes of ventilation were not compared in our population. Further studies are needed to clarify whether volume-targeted ventilation is indeed effective in achieving optimal pCo2.

In patients ventilated alone, we focused on three respiratory parameters (mechanical VT, total RR, and minute ventilation) throughout the treatment period and found a reduction in respiratory rate, tidal volume, and minute ventilation (Figure 1). This could be due to a reduction in metabolic rate, oxygen consumption, and carbon dioxide production during TH, as well as an improvement in lung function.

Although there are no treatment options to prevent hypocapnia in spontaneously breathing patients, non-ventilated infants in our study did not show increased RR or hypocapnia. This confirms and supports the decision to avoid elective intubation during TH.

pO2 values were within the normal range in the entire study population.

Logistic regression analysis shows that the risk factors for mechanical ventilation were intrapartum sentinel events, maternal infectious disease, 5-min Apgar score, and hypotension. In our population, the need for mechanical ventilation and blood gas levels during TH had no effect on short-term neurologic outcomes. Although ventilated patients with poorer biochemical and clinical birth data had a higher percentage of abnormalities for basal ganglia, thalami, PLIC, and cortex in the magnetic resonance score, no statistically significant differences in predictive lesions for abnormal outcomes were found between the two groups.

## Conclusion

In summary, this study shows that individualized ventilatory management during TH, which does not necessarily include mechanical ventilation, can ensure stable blood gas levels. Individualized ventilatory management does not appear to affect short-term neurological outcomes. In infants requiring mechanical ventilation, volume-dependent ventilation may help maintain stable blood gas levels.

Tailored ventilation management, using mechanical ventilation only when needed and volume-controlled ventilation, may therefore be the optimal strategy during TH.

Follow-up data from this population are needed to confirm these findings with regard to long-term neurological development.

## Data Availability

The raw data supporting the conclusions of this article will be made available by the authors, without undue reservation.
